# The Burden of Diabetic Foot Ulcers on Hospital Admissions and Costs in Romania

**DOI:** 10.3390/jcm14041248

**Published:** 2025-02-13

**Authors:** Adriana Rusu, Gabriela Roman, Bogdan Stancu, Cornelia Bala

**Affiliations:** 1 Department of Diabetes and Nutrition Diseases, “Iuliu Hatieganu” University of Medicine and Pharmacy, 400012 Cluj-Napoca, Romania; adriana.rusu@umfcluj.ro (A.R.); groman@umfcluj.ro (G.R.); cbala@umfcluj.ro (C.B.); 2 Diabetes Center, Emergency Clinical County Hospital Cluj, 400006 Cluj-Napoca, Romania; 3 Second Department of Surgery, “Iuliu Hatieganu” University of Medicine and Pharmacy, 400012 Cluj-Napoca, Romania; 4 Second Department of Surgery, Emergency Clinical County Hospital Cluj, 400006 Cluj-Napoca, Romania

**Keywords:** diabetes, foot ulcers, costs, length of hospitalization, in-hospital care

## Abstract

**Background/Objectives**: Diabetic foot ulcers represent an important economic burden for healthcare systems worldwide. We aimed to analyze the costs of care for diabetic foot ulcers (DFUs) associated with peripheral neuropathy (DPN) and peripheral arterial disease (PAD) and their trends in a tertiary-care hospital. **Methods**: We used data from the electronic system of the Emergency Clinical County Hospital Cluj-Napoca, Romania. We included all episodes of hospitalization with a discharge date between 1 January 2015 and the 31 December 2018 and a discharge diagnosis of type 1 or type 2 diabetes, DPN, PAD, and foot ulcers. **Results**: During the analyzed period, 1094 episodes of hospitalization with type 1 or type 2 diabetes and DFUs were recorded. Of these, 25.9% had neuropathic, 47.1% ischemic, and 16.6% neuroischemic DFUs. The median length of hospital stay was 8.0 days, and the median cost per episode of hospitalization was 810.8 EUR, with no significant variations during the analyzed years. The cost per episode of hospitalization was higher in cases with ischemic DFUs than for other etiologies of foot ulcers for 2015–2017 (*p* < 0.001). In 2018, the costs for ischemic and neuroischemic DFUs were similar and significantly higher compared to neuropathic ones. Predictors of higher costs per episode of hospitalization included the etiology of the DFUs (β = 0.032, *p* = 0.034) and the length of hospital stay (β = 0.860, *p* < 0.001). **Conclusions**: By analyzing data from a tertiary care hospital, we showed higher costs for the care of persons with ischemic DFU.

## 1. Introduction

Diabetes represents a serious burden for healthcare systems and society. According to the most recent IDF estimates, 537 million people were living with diabetes in 2021, and it is projected to reach 783 million by 2045 [1]. Increasing economic costs of diabetes have already been reported all around the globe. Worldwide, health expenditures due to diabetes increased from 232 billion USD in 2007 to 966 billion USD in 2021, representing a 316% increase in costs; furthermore, diabetes costs are expected to reach 1.05 trillion USD by 2045 [1]. Data on diabetes-related health expenditure for Romania are also available from the IDF; according to the 10th Diabetes Atlas, the total yearly expenses at the country level for adults (20–79 years of age) are ranked in six categories, and Romania is included in the second category (with total expenses of 1–10 billion USD in 2021, while diabetes-related health expenditure per person with diabetes was in the lowest range, with an average of 930.2 USD/person/year as compared to the top 10 countries for diabetes-related health expenditure per person with diabetes (>6500 USD)) [1].

Diabetic foot ulcers (DFUs) represent an important source of morbidity and mortality due to the risk of amputations and repeated hospital admissions and an important economic burden for healthcare systems and governments around the world. In persons with diabetes, the lifetime risk of developing a foot ulcer ranges between 15% and 25% [2], while the risk of recurrence reaches 50% [3]. In 2016, 131 million people had diabetes-related lower extremity complications, resulting in 16.8 million years lived with disability, including 2.5 million from foot ulcers [4]. It has been estimated that up to 33% of the costs of diabetes are related to diabetic foot complications and include direct costs (hospital admission, antibiotics, dressings, and surgery) and indirect costs (sick leaves, reduced work productivity) [5]. Following the diagnosis of a DFU, there is a five-fold increase in expenses during the first year, the costs increasing with the severity of the DFU; repeated admissions and emergency room presentations account for a major percentage of these expenditures [6].

In Romania, little is known about the costs associated with the care of DFUs. Only two previously published papers assessed the costs associated with DFUs in hospitalized patients. These studies showed an increase in expenses/episode of hospitalization of patients with a DFU from 724 EUR in 2012–2017 to 810.9 EUR in 2015–2018 [7,8]. Given the increasing trend in the prevalence of diabetes in Romania, a longer life expectancy with diabetes, and a longer diabetes duration, an increasing economic burden of diabetes is expected. Since foot complications represent a major source of costs among persons with diabetes, and hospital admission accounts for the largest percentage of the direct costs of DFUs [5], our objective was to investigate the predictors of costs for in-hospital care of these two factors. Building upon our previous study [8], here we aimed to comparatively analyze the costs of the care of neuropathic and ischemic and neuroischemic DFUs and their trends over five years in a tertiary-care hospital.

## 2. Materials and Methods

### 2.1. Data Source and Study Sample

The data source and study sample of this retrospective database analysis have been previously described [8]. Briefly, we used data from the electronic system of the Emergency Clinical County Hospital Cluj-Napoca, Romania. This hospital functions as both a tertiary and a secondary care hospital. It provides healthcare services for adult patients from Cluj-Napoca and the surrounding areas (>500,000 inhabitants) and also for adult patients from the whole Transylvania region (a population of > 5.4 million inhabitants) for severe health issues that cannot be addressed in the local secondary care hospitals—including revascularization procedures. The average number of discharges per year in the 2015–2018 period was >56000 cases, and other figures for the entire hospital are publicly available online [9]. Thus, analyzing the data from the Emergency Clinical County Hospital Cluj provides a good overview of DFU-associated costs in both secondary and tertiary care hospitals.

For this analysis, we included all episodes coded as hospitalizations for all adult patients (≥18 years of age) with a discharge date between 1 January 2015 and 31 December 2018 and a primary or secondary diagnosis at the discharge of type 1 or type 2 diabetes (International Statistical Classification of Diseases and Related Health Problems 10th Revision [ICD-10] codes E10 and E11), peripheral diabetic neuropathy (DPN) (ICD-10 codes E10.40–E10.42, E10.49, E11.40–E11.42, E11.49, G62.8, G62.9, and G63.3), peripheral arterial disease (PAD) (ICD-10 codes E10.51, E10.52, E11.51, E11.52, I70.20–I70.25, and I73.8) and foot ulcers (ICD-10 codes E10.73, E11.73, E10.52, E11.52, I70.23, I70.24, I70.25, and L97). Cases were grouped according to the presence of DPN and/or PAD; when the discharge diagnosis specified the presence of foot ulcers and DPN, the case was coded as neuropathic DFU; when the discharge diagnosis specified the presence of foot ulcers and PAD, the case was coded as ischemic DFU; and when the discharge diagnosis specified the presence of foot ulcers and both DPN and PAD, the case was coded as neuroischemic DFU. When neither DPN nor PAD was present among the discharge diagnoses, the etiology of foot ulcers was considered as not specified. As no protocol was in place in the institution for ascertaining the etiology of foot ulcers in patients with diabetes in the studied period, we cannot exclude the existence in these patients of either DPN or PAD, and thus they were included in the analysis as “no specified etiology”.

Patient-level data on age, sex, number of episodes of hospitalization, length of hospitalization for each episode, and total direct inpatient care costs/episode of hospitalization were retrieved from the electronic system for all cases analyzed and exported by the information technology service provider of the institution without any personal identifiers. Each participant received a code that allowed identification in the database. As previously described [8], readmission was treated as a new episode of hospitalization, and an episode of hospitalization was defined as the time from patients’ admission to the hospital until discharge or death.

The study protocol was approved by the Ethics Committee of the Emergency Clinical County Hospital Cluj-Napoca, Romania (protocol version 1.0 dated 15 April 2019 and date of approval 29 May 2019). The informed consent was waived due to retrospective database analysis.

### 2.2. Costs of Hospitalization

Data used for the analysis of costs/episode of hospitalization were the ones available at discharge, as listed in the hospital electronic system, irrespective of their reimbursement status and including the cost of bed usage (hospitalization), food allowance, and the cost of drugs, medical supplies, medical devices, and investigations and procedures (laboratory and imagistic investigations and surgical procedures performed). The costs of bed usage were calculated as the cost/day of hospitalization multiplied by the number of days of hospitalization. Food allowance was calculated as the daily food allowance multiplied by the number of days of hospitalization.

As previously described, public hospital funding in Romania is a mix of prospective and retrospective reimbursement based on the Australian Diagnosis Related Groups (DRG) system [10]. The costs/day of hospitalization for bed usage are in a fixed amount set yearly by the Ministry of Health and the National Health Insurance House according to the type of hospital and hospital department and are based on the severity of cases in the previous year. The daily food allowance is also set to a fixed amount for all public hospitals and varies according to department type. In addition, the costs of drugs purchased by the hospitals are regulated by the Ministry of Health through direct negotiation, with marketing authorization holders and a claw-back tax aiming to control drug expenditures [11]. For medical supplies, medical devices, investigations, and procedures, public hospitals do not use established unit costs per unit at the patient level; an average cost is used and is calculated by dividing the total costs by the number of services performed or products used [12,13,14]. The above costs are automatically calculated and reported at each patient’s discharge.

All cost data were obtained from the electronic system of the Emergency Clinical Hospital Cluj in the Romanian Leu. To account for the inflation during the 2015–2018 period, all costs were adjusted for the annual inflation rates using the Consumer Price Index (CPI) provided by the Romanian National Statistics Institute (Bucharest, Romania) [15]. We chose 2018 as the base year for inflation adjustment. Subsequently, costs were converted to EUR, considering the average exchange rate provided by the Romanian National Bank for each year of the period 2015–2018 [16].

### 2.3. Statistical Analysis

Total CPI-adjusted costs for hospitalizations and CPI-adjusted median cost per episode of hospitalization were reported for the whole sample and by groups with neuropathic, ischemic, and neuroischemic DFUs and with no specified etiology, and stratified per year, sex, age groups, and type of diabetes. Statistical analysis was performed using SPSS version 26 (SPSS Inc., Chicago, IL, USA), and data are presented as median (quartile 1; quartile 3) and number (percentage). The variables were tested for normality using the Shapiro–Wilk test. The comparison between groups was performed using the Mann–Whitney U and Kruskal–Wallis tests (for the comparison of two or three or more groups, respectively) and chi-square tests. Predictors of length of hospital stay and median CPI-adjusted costs per episode of hospitalization were identified using multivariate linear regression. For this regression analysis, CPI-adjusted costs and the number of days of hospitalization were logarithmically transformed and used as dependent variables. A *p*-value of *<* 0.05 was considered statistically significant.

## 3. Results

### 3.1. Study Sample

Between 1 January 2015 and 31 December 2018, 28,055 episodes of hospitalization for 16,868 patients with diabetes were recorded in the Emergency Clinical County Hospital Cluj [8]. Of these, 1094 (3.9%) had type 1 or type 2 diabetes, with a primary or secondary diagnosis of foot ulcers. Those with a specific form of diabetes were excluded from this analysis due to the limited number (eight cases during the analyzed period). A total of 1086 (99.3%) cases were discharged alive. The median age of the analyzed patients was 65.0 years; the majority were men (71.6%) and had type 2 diabetes (93.5%; Table 1).

According to etiology, 25.9% of the episodes of hospitalizations for DFUs were neuropathic, 47.1% ischemic, and 16.6% neuroischemic, with a decreasing trend for neuropathic and neuroischemic DFUs and an increasing one for the ischemic ones during the analyzed period (*p* for trend *<* 0.001). In 10.4% of the cases, the etiology was not specified in discharge diagnoses. Those with ischemic and neuroischemic DFUs were older (median age 67.0 years and 65.0 years vs. 62.0 years and 64.0 years, *p <* 0.001) than those with neuropathic DFUs and those with no specified etiology. Also, those with ischemic DFUs had more frequent type 2 diabetes (97.3% vs. 90.7% vs. 86.9%, *p <* 0.001) and were more frequently women (31.7% vs. 17.6% vs. 23.7%, *p <* 0.001) than those with neuroischemic and neuropathic DFUs. The frequency of men was higher in the group with neuroischemic DFUs (82.4%).

### 3.2. Length of Hospital Stay for Foot Ulcers

The median length of the hospital stay was 8.0 days, with no statistically significant differences between analyzed years (*p* = 0.075). Length of hospital stay was significantly longer in women than in men (9.0 days vs. 8.0 days, *p* = 0.017). No difference was observed in the length of hospital stay according to the type of diabetes (*p* = 0.126) or age group (*p* = 0.379) when data from all years were analyzed together. No statistically significant difference between the study years was observed for the length of hospital stays when data were analyzed according to sex, age group, and the type of diabetes (Supplementary Table S1).

**Figure 1:**
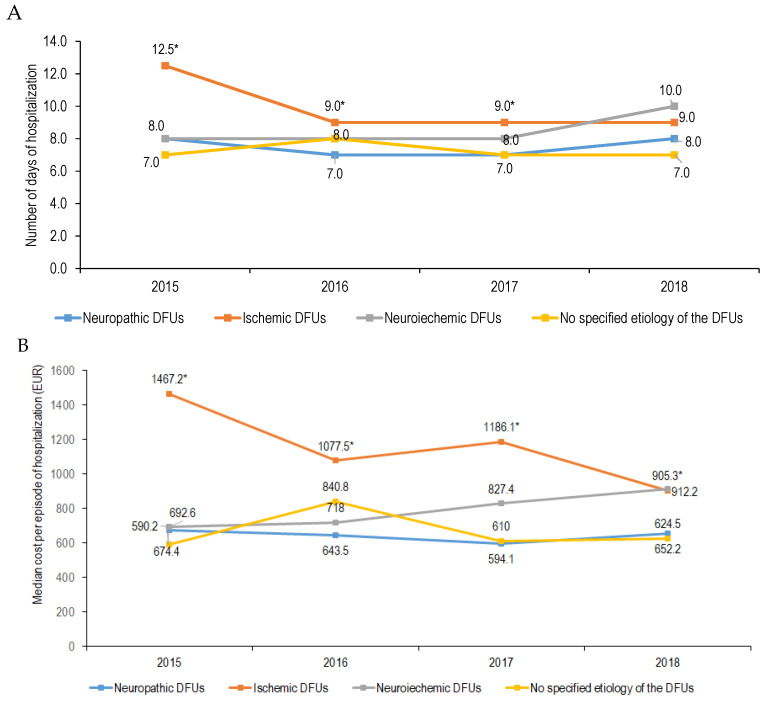
Median length of hospital stay (panel (**A**)) and cost per episode of hospitalization (panel (**B**)) by foot ulcer etiology. * Statistically significant difference (*p* < 0.05, Kruskal–Wallis test). DFUs, diabetic foot ulcers.

In cases with ischemic DFU, the median length of hospital stay was 10.0 days, reaching a maximum in 2015 (12.5 days) and decreasing thereafter to 9.0 days (*p* for trend = 0.003; Figure 1A). A similar evolution was seen in the length of hospital stay in patients with type 2 diabetes and those ≥65 years of age and with ischemic DFUs (*p* for trend = 0.004 and 0.07, respectively). No significant change in the length of hospital stay was observed over time in women or any age group (*p* for trend > 0.05 for all). Also, no statistically significant difference in the length of hospital stay was observed between diabetes types, sex, and age groups (*p* > 0.05 for all; Table 2).

In cases with neuropathic DFUs, the length of hospital stay was 7.0 days, with a maximum of 8.0 days in 2015 and 2018 (*p* for trend = 0.042; Figure 1A). No significant change in the length of hospital stay was observed over time in type 1, women or men, or any age group (*p* for trend > 0.05 for all). However, in type 2 diabetes, it decreased significantly from 8.0 days in 2015 to 7.0 days in 2016 and 2017 and increased again in 2018 (8.0 days, *p* = 0.050). Additionally, a longer length of stay was observed in women with neuropathic DFUs than in men (*p* = 0.001; Table 2).

In cases with neuroischemic DFUs, the length of hospital stay was 8.0 days, with no statistically significant difference between studied years (*p* = 0.596; Figure 1A). Also, no statistically significant difference in the length of hospital stay was observed between diabetes types, sex, and age groups (*p* > 0.05 for all; Table 2).

In cases with no specified etiology of the DFU, the length of hospital stay was 7.0 days, with no statistically significant difference between studied years (*p* = 0.200; Figure 1A). No significant variation in the length of hospital stay was observed over time in type 1 or type 2 diabetes, men, or any age group (*p* > 0.05 for all). Also, no statistically significant difference in the length of hospital stay was observed between diabetes types, sex, and age groups (*p* > 0.05 for all; Table 2).

Overall, the length of hospital stay was significantly longer in cases with ischemic DFUs than in those with neuropathic or neuroischemic DFUs (10.0 days vs. 7.0 days vs. 8.0 days, *p <* 0.001). By the year analyzed, the difference remained statistically significant for 2015–2017. However, in 2018, the length of hospital stay was similar irrespective of etiology (*p* = 0.397; Figure 1A).

In the multivariate linear regression analysis, neither age, sex, type of diabetes, or the etiology of the foot ulcers were predictors of the length of hospital stay (*p* > 0.05; Table 3).

### 3.3. Cost of Hospitalization During the Analyzed Period for Foot Ulcers

The total CPI-adjusted costs for the in-hospital care of patients with DFUs between 2015 and 2018 were 1,270,157.5 EUR. The total expenses for the hospital care of persons with diabetes in the analyzed period were 26,418,126.8 EUR. In relation to this figure, the expenses for the hospitalization of patients with DFUs represented 4.8%. During the period analyzed, the total yearly costs had a decreasing trend from 344,133.3 EUR in 2015 to 312,294.8 EUR in 2016, 307,047.4 EUR in 2017, and 306,682.1 EUR in 2018, in parallel with a decrease in the number of episodes of hospitalization for foot ulcers (from 280 in 2015 to 263 in 2018).

The median CPI-adjusted cost per episode of hospitalization for DFUs was 810.8 EUR, with no significant variation during the analyzed years (*p* for trend = 0.332). The cost per episode of hospitalization was higher in type 2 diabetes than in type 1 diabetes (*p* = 0.002), in women than in men (*p* = 0.001), and in younger age groups (max 1031.5 EUR in 18–40 years, min 747.6 EUR in 40–65 years age group). No statistically significant difference between the study years was observed for the cost per episode of hospitalization when data were analyzed according to sex, age group, and type of diabetes (Supplementary Table S2).

In cases with ischemic DFUs, the CPI-adjusted costs of all episodes of hospitalization during the analyzed period were 788,539.7 EUR. The median cost per episode of hospitalization was 1168.6 EUR, reaching a maximum in 2015 (1467.2 EUR) and decreasing thereafter to 905.3 EUR in 2018 (*p* for trend = 0.001; Figure 1B). A similar evolution was seen in the cost per episode of hospitalization in patients with type 2 diabetes and ischemic DFUs (*p* for trend = 0.001), in both women and men (*p* = 0.021 and 0.023, respectively) and those >65 years of age (*p* = 0.007). No significant change in the cost per episode of hospitalization was observed over time in type 1 diabetes and younger age groups (*p* for trend > 0.05 for all). Also, no statistically significant difference in the costs was observed between sexes and age groups (*p* > 0.05 for all). However, the costs of hospitalization for patients with type 2 diabetes and DFUs were significantly higher as compared to those with type 1 diabetes (Table 4).

In cases with neuropathic DFU, the CPI-adjusted costs of all episodes of hospitalization during the analyzed period were 194,610.5 EUR. The median cost per episode of hospitalization was 633.3 EUR, with no significant variations during the analyzed period (*p* for trend = 0.152; Figure 1B). No significant changes in the cost per episode of hospitalization for foot ulcers were observed over time in type 1 or type 2 diabetes, women, men, or any age group (*p* for trend > 0.05 for all). Overall, a higher cost per episode of hospitalization was observed in women with neuropathic DFUs than in men (*p* = 0.001), and the difference was due to significantly higher costs per episode of hospitalization in women in 2015 and 2016 (Table 4).

In cases with neuroischemic DFUs, the CPI-adjusted costs of all episodes of hospitalization during the analyzed period were 185,631.3 EUR. The median cost per episode of hospitalization was 731.5 EUR, with no significant variations during the analyzed period (*p* for trend = 0.289; Figure 1B). No significant changes in the cost per episode of hospitalization for DFUs were observed over time in type 1 or type 2 diabetes, women, men, or any age group (*p* for trend > 0.05 for all). Also, the cost per episode of hospitalization was similar in men and women, in type 1 and type 2 diabetes, and in all age groups (*p* > 0.05 for all; Table 4).

In cases with no specified etiology of the DFUs, the cost of all episodes of hospitalization was 101,376.05 EUR. The median cost per episode of hospitalization was 653.3 EUR, with no statistically significant difference between the studied years (*p* = 0.056; Figure 1B). When data were analyzed according to study groups, a significant variation in the costs was observed across the studied years in type 2 diabetes and women. No significant variation in the costs per episode of hospitalization was observed between men and women, type of diabetes, or age group over time in type 1 or type 2 diabetes, men, or any age groups (*p* > 0.05 for all; Table 4).

Overall, the costs per episode of hospitalization were significantly higher in ischemic DFUs than in those with neuropathic, neuroischemic, and no specified etiology of the DFUs (1168.6 EUR vs. 633.3 EUR vs. 731.5 EUR vs. 653.3 EUR, *p <* 0.001). The difference in costs between ischemic and other DFU types remained statistically significant for 2015–2017 (*p <* 0.001). In 2018, the costs for ischemic and neuroischemic DFUs were similar and significantly higher compared to neuropathic and no-specified etiology foot ulcers (Figure 1B).

In the multivariate linear regression analysis, the predictors of higher cost per episode of hospitalization were the etiology of the DFUs (standardized β = 0.032, *p* = 0.034) and the length of hospital stay (standardized β = 0.860, *p <* 0.001). Sex, age, and type of diabetes were not associated with the costs (Table 3).

## 4. Discussion

In this analysis of data derived from a large tertiary hospital in Romania, we analyzed the temporal trends of admission for DFUs, the costs associated with their in-hospital care, and the main drivers of these costs. During the period analyzed, the burden represented by the in-hospital care of DFUs remained high, representing 4% of the hospitalization episodes in patients with diabetes. Overall, the frequency of cases slightly decreased; a temporal decreasing trend for the admission of neuropathic and neuroischemic DFUs, and an increasing trend for the ischemic ones, was observed. The total costs of hospitalization of patients with DFUs were also significant, exceeding 1.2 million EUR and representing around 5% of the hospital’s expenses in persons with diabetes. During the period analyzed, we observed a decreasing trend in the yearly costs of hospitalization for DFUs between 2015 and 2018. This decrease occurred in the context of a slight decrease in the number of episodes of hospitalization and a decrease in the costs of hospitalization for ischemic DFUs. Our results complement previously published data on patients with diabetes in Romania [7,8]. Of note, in our study, the neuropathic DFUs accounted for less than half of the foot ulcer cases hospitalized in the period analyzed, lower than in other reports [17]. This may be due to an effort to control costs by treating ambulatory, less severe cases with neuropathic DFUs, and this may have decreased the total and yearly costs of hospitalization of analyzed cases.

The median CPI-adjusted cost per episode of hospitalization for DFUs in our study was 810.8 EUR, stable during the analyzed years and significantly higher as compared to those previously reported in patients with diabetes without foot ulcers in Romania (587.1 EUR) [8]. Higher costs of the in-hospital care of DFUs, as compared to the in-hospital care of diabetic patients without foot ulcers or non-diabetic foot ulcers, have been consistently reported in numerous studies around the world and across multiple healthcare systems. In a study performed in a tertiary hospital in Turkey, DFUs doubled the cost of the in-hospital care of patients with diabetes (976.1 USD in DFUs vs. 430.3 USD in patients with diabetes and without foot ulcers) [18]. A multicenter study performed in seven hospitals in the Greater Toronto Area, Canada from 2010 to 2015 showed almost three times higher costs of care for DFU-related admissions when compared to non-diabetic foot ulcers [19]. In a systematic review of the literature on the costs of different chronic ulcers across the world, Chan et al. calculated a median cost per episode of hospitalization of 18,349 USD for DFUs vs. 13,393 USD for pressure ulcers (adjusted to 2015 USD) [20].

Regarding Romania, the costs associated with the care of DFUs have been insufficiently addressed. Only one publication is available and reports data originating from the same tertiary hospital. In this study, Sima et al. [7] compared patients admitted between 2012 and 2017 in the diabetes clinic of this tertiary hospital and showed higher costs of care for DFUs as compared to patients with diabetes and no foot ulcers (724 EUR vs. 517 EUR). The costs we report are higher as compared to those reported by Sima et al. [7] in our hospital and may be due to the period included in the analysis, adjustment for the inflation rate, and the inclusion of data for patients with ulcerations treated in surgery departments or with foot revascularization due to PAD and traditionally associated with higher costs.

As compared to other countries, the average cost per episode of hospitalization was significantly lower in our country. Our results may be explained by the lower costs/day of hospitalization for bed usage and food allowance, lower costs of investigations during the hospital stay, lower wages of medical staff, and drug costs, which are regulated by the Ministry of Health and the National Health Insurance House [8], as described in the Section 2. To put these lower costs per episode of hospitalization for DFUs in the context of the global costs for healthcare, total spending for healthcare in Romania is only 1663 EUR per capita per year as compared to the EU average of 4028 EUR [21]. However, the costs of care vary across countries and regions and are higher in high-income countries as compared to middle- and low-income countries [22]. We acknowledge a limited generalizability of data provided here to other countries given the differences regarding healthcare-associated costs and management. However, due to these differences, epidemiological and health economic studies across multiple countries are difficult to perform, and, thus, data from individual countries reporting local costs of care are the ones providing an overview of the healthcare burden of DFUs.

The etiology behind the DFUs influences their associated costs. We found significantly higher costs associated with ischemic DFUs than for neuropathic DFUs. The costs of all episodes of hospitalization for ischemic and neuroischemic DFUs were five times higher as compared to the neuropathic ones (788,539.7 EUR for ischemic and 185,631.3 EUR for neuroischemic vs. 194,610.5 EUR for neuropathic DFUs). Furthermore, the cost per episode of hospitalization was almost double in cases with ischemic DFUs than in those with neuropathic DFUs (1168.6 EUR vs. 633.3 EUR). Higher costs of therapy for ischemia-associated DFUs have also been reported in other countries and healthcare systems. In an Italian cohort originating from a tertiary care hospital, Da Ros et al. [23] showed an 18% increase in the cost per episode of hospitalization when ischemia was associated with the infection of DFUs vs. infection alone (7657 EUR vs. 6486 EUR). Similarly, analyzing seven years of data from a single hospital in China, Lu et al. [24] showed significantly higher median costs of care for ischemic DFUs than for those without ischemia. In a study based on medical claims in the US from 2000 and 2001, including outpatients and inpatients with or without PAD, the average cost per DFU was 5218 USD for patients without PAD and 23,372 USD for patients with PAD [25].

The main driver of the cost of hospitalization, in addition to the etiology of DFUs (i.e., PAD), in our study, as in previous ones [23], was the length of hospital stay. Indeed, the longer length of hospital stay for ischemic DFUs observed in our study might explain the higher costs of hospitalization associated with this diagnosis. Irrespective of the discharge diagnosis, the length of hospital stay is one of the major drivers of the hospitalization costs in patients with diabetes in Romania and elsewhere [8,26]. Furthermore, it has been calculated that, in persons with diabetes, each day of hospital stay increases the costs by 3% for type 1 diabetes and by 2% for type 2 diabetes, thus having an incremental effect on the costs [26]. However, these are only the drivers that we could analyze. We cannot exclude other drivers, such as investigations and procedures as illustrated by the significantly higher cost of the in-hospital care of ischemic DFUs than of neuropathic DFUs in 2018, with a similar length of hospital stay.

There are several limitations to this study. First, only the costs of persons discharged with a diagnosis of diabetes were available for this analysis. Thus, a comparison with inpatient costs of care for foot ulcers in persons without diabetes was not possible. Another limitation is linked to the data included in the database, leading to the unavailability of cost split per medication, investigations and procedures performed (e.g., wound debridement, revascularization), hospitalization, and food allowance. The availability of data from a single center is another study limitation. Although our hospital functions as both a secondary and tertiary care hospital, the costs depicted could overestimate the costs associated with diabetes care in other secondary care hospitals. Furthermore, the availability of sociodemographic characteristics of participants, the reason for patient admission, the proportion of infected diabetic foot ulcers, and the DFU classification from our data (such as Texas, WiFi, and Wagner) may have provided a deeper insight into the figures found.

## 5. Conclusions

In conclusion, by analyzing data from a tertiary care hospital, we showed the costs associated with the public hospital care of persons with DFUs and their main drivers, identifying ischemia as the etiology with an increasing frequency and associated with higher costs. To the best of our knowledge, this is the first study to investigate the economic burden correlated with ischemic and neuropathic DFU care in Romania. Considering the burden that diabetes already represents for the Romanian healthcare system and the foreseen increase in foot ulcers in the context of increasing diabetes prevalence, this study allows for the planning of financial resources allocated for the screening and care of foot problems and could also be of value for countries with similar healthcare systems and cost structures.

## Figures and Tables

**Table 1:** Description of the study sample (episodes of hospitalization).

	All Sample N = 1094	2015 N = 280	2016 N = 273	2017 N = 278	2018 N = 263	*p*-Value for Trend
Type of diabetes, n (%)						0.853
Type 1	71 (6.5%)	19 (6.8%)	19 (7.0%)	19 (6.8%)	14 (5.3%)	
Type 2	1023 (93.5%)	261 (93.2%)	254 (93.0%)	259 (93.2%)	249 (94.7%)	
Age, years *	65.0	64.0	65.0	65.0	67.0	0.269
	(59.0; 72.0)	(59.0; 71.0)	(59.0; 71.0)	(59.0; 73.0)	(60.0; 72.0)	
Age groups, n (%)						0.063
18–40 years	10 (0.9%)	4 (1.4%)	1 (0.4%)	3 (1.1%)	2 (0.8%)	
40–65 years	502 (45.6%)	138 (49.3%)	135 (49.5%)	127 (45.7%)	99 (37.6%)	
≥65 years	590 (53.5%)	138 (49.3%)	137 (50.2%)	148 (53.2%)	162 (61.6%)	
Men, n (%)	783 (71.6%)	202 (72.1%)	189 (69.2%)	210 (75.5%)	182 (69.2%)	0.301
Foot ulcer etiology, n (%)						<0.001
Neuropathic	283 (25.9%)	91 (32.5%)	63 (23.1%)	86 (30.9%)	43 (16.3%)	
Ischemic	515 (47.1%)	116 (41.4%)	126 (46.2%)	123 (44.2%)	150 (57.0%)	
Neuroischemic	182 (16.6%)	59 (21.1%)	46 (16.8%)	36 (12.9%)	41 (15.6%)	
Not specified etiology	114 (10.4%)	14 (5.0%)	38 (13.9%)	33 (11.9%)	29 (11.0%)	

N/n (%), number (percentage) of episodes of hospitalizations. * Presented as median (quartile 1; quartile 3). The Kruskal–Wallis and chi-square tests were used for the calculation of the *p*-values.

**Table 2:** Median length of stay per episode of hospitalization by etiology of foot ulcers.

	**All Sample** **N = 1094**	**2015** **N = 280**	**2016** **N = 273**	**2017** **N = 278**	**2018** **N = 263**	***p*** **-Value for Trend**
Overall cases with foot ulcers	8.0 (6.0; 12.0)	9.0 (7.0; 13.0)	8.0 (6.0; 12.0)	8.0 (6.0; 11.0)	8.0 (6.0; 12.0)	0.075
Neuropathic DFUs						
Type of diabetes						
Type 1	7.0 (6.0; 9.0)	8.0 (7.0; 8.5)	7.0 (6.0; 9.0)	7.0 (6.0; 8.5)	7.0 (6.5; 9.0)	0.867
Type 2	7.0 (6.0; 9.0)	8.0 (6.0; 10.0)	7.0 (6.0; 10.0)	7.0 (6.0; 8.0)	8.0 (6.5; 10.0)	0.050
*p*-value	0.915	0.893	0.909	0.660	0.595	
By sex						
Women	9.0 (6.5; 11.5)	10.0 (7.0; 12.0)	8.5 (6.0; 13.0)	7.0 (7.0; 9.0)	7.5 (6.0; 10.0)	0.343
Men	7.0 (6.0; 9.0)	7.0 (6.0; 9.0)	7.0 (6.0; 9.0)	7.0 (6.0; 8.0)	8.0 (7.0; 10.0)	0.084
*p*-value	0.001	0.005	0.035	0.261	0.724	
By age groups						
18–40 years	7.0 (6.0; 11.0)	7.0 (6.5; 10.5)	11.0	4.0	-	0.344
40–65 years	7.0 (6.0; 10.0)	8.0 (7.0; 10.0)	7.0 (6.0; 9.0)	7.0 (6.0; 9.0)	8.0 (7.0; 10.0)	0.101
≥65 years	7.0 (6.0; 10.0)	8.0 (6.0; 10.0)	6.0 (5.0; 9.0)	7.0 (6.0; 8.0)	8.0 (6.0; 10.0)	0.406
*p*-value	0.829	0.972	0.232	0.369	0.483	
Ischemic DFUs						
Type of diabetes						
Type 1	7.0 (6.0; 11.0)	15.0 (11.0; 16.5)	6.0 (4.0; 7.0)	8.0	13.0 (11.0; 15.0)	0.050
Type 2	10.0 (6.0; 15.0)	12.0 (8.0; 18.0)	9.0 (7.0; 14.0)	9.5 (6.0; 14.0)	9.0 (6.0; 14.0)	0.004
*p*-value	0.207	0.844	0.010	0.780	0.346	
By sex						
Women	10.0 (6.0; 15.0)	13.0 (8.5; 17.5)	10.0 (6.0; 15.0)	8.0 (5.0; 16.0)	9.0 (6.0; 12.0)	0.060
Men	10.0 (6.0; 14.0)	11.5 (7.0; 18.0)	8.0 (6.0; 13.0)	10.0 (7.0; 13.5)	9.0 (5.0; 14.0)	0.047
*p*-value	0.601	0.624	0.282	0.500	0.945	
By age groups						
18–40 years	13.0 (8.0; 18.0)	-	-	8.0	18.0	1.000
40–65 years	10.0 (6.0; 15.5)	12.5 (7.0; 22.0)	9.0 (6.0; 14.0)	8.0 (6.0; 12.0)	8.0 (6.0; 14.0)	0.092
≥65 years	10.0 (6.0; 14.0)	12.5 (8.0; 16.0)	9.0 (7.0; 13.5)	10.0 (6.0; 14.0)	9.0 (5.0; 13.5)	0.047
*p*-value	0.860	0.527	0.907	0.784	0.566	
Neuroischemic DFUs						
Type of diabetes						
Type 1	8.0 (7.0; 11.0)	7.0 (6.0; 8.0)	8.0 (6.0; 8.5)	10.0 (7.0; 13.0)	11.0 (9.5; 11.5)	0.295
Type 2	8.0 (7.0; 11.0)	8.0 (6.0; 11.0)	8.0 (7.0; 11.0)	8.0 (6.0; 12.0)	10.0 (7.0; 12.0)	0.821
*p*-value	0.934	0.359	0.496	0.548	0.584	
By sex						
Women	8.0 (7.0; 12.5)	7.0 (5.0; 8.0)	10.0 (7.0; 21.0)	8.0 (8.0; 9.0)	11.0 (7.0; 15.0)	0.359
Men	8.0 (7.0; 11.0)	8.0 (7.0; 11.0)	8.0 (7.0; 10.0)	8.0 (6.0; 13.0)	9.5 (7.0; 11.0)	0.862
*p*-value	0.836	0.181	0.238	1.000	0.463	
By age groups						
18–40 years	7.0	7.0	-	-	-	-
40–65 years	8.0 (7.0; 11.0)	8.0 (6.5; 10.5)	8.0 (7.0; 10.0)	8.0 (7.0; 12.0)	11.0 (8.5; 12.0)	0.311
≥65 years	8.0 (6.5; 11.0)	8.0 (6.0; 11.0)	7.0 (7.0; 11.0)	8.0 (6.5; 12.5)	9.5 (7.0; 11.0)	0.939
*p*-value	0.821	0.864	0.876	0.754	0.204	
No specified etiology of the DFUs						
Type of diabetes						
Type 1	12.0 (9.0; 19.5)	-	-	12.0	16.5 (6.0; 27.0)	1.000
Type 2	7.0 (7.0; 10.0)	7.0 (6.0; 8.0)	8.0 (7.0; 12.0)	7.0 (6.0; 10.5)	7.0 (7.0; 9.5)	0.171
*p*-value	0.338	-	-	0.303	0.650	
By sex						
Women	7.0 (7.0; 10.0)	7.0 (6.0; 7.0)	10.0 (8.0; 13.5)	7.0 (7.0; 11.5)	7.0 (6.5; 9.5)	0.008
Men	7.0 (6.0; 10.0)	8.0 (6.0; 9.5)	7.0 (7.0; 10.0)	7.0 (6.0; 10.0)	7.0 (7.0; 11.0)	0.906
*p*-value	0.302	0.209	0.044	0.166	0.845	
By age groups						
18–40 years	19.0 (11.0; 27.0)	-	-	11.0	27.0	1.000
40–65 years	7.0 (7.0; 10.5)	7.0 (7.0; 8.0)	8.0 (7.0; 13.0)	7.0 (7.0; 10.5)	7.0 (7.0; 10.0)	0.723
≥65 years	7.0 (6.0; 10.0)	6.5 (5.5; 8.5)	8.5 (7.0; 11.0)	7.0 (6.0; 10.0)	7.0 (6.0; 7.5)	0.167
*p*-value	0.125	0.755	0.675	0.455	0.134	

N, number of episodes of hospitalizations; DFU, diabetic foot ulcer. The Kruskal–Wallis and Mann–Whitney tests were used for the calculation of the *p*-values.

**Table 3:** Predictors of length of stay and costs per episode of hospitalization.

Predictor	Length of Stay per Episode of Hospitalization		Adjusted Average Costs per Episode of Hospitalization	
	Standardized β Coefficient	*p*-Value	Standardized β Coefficient	*p*-Value
Age, years	−0.019	0.552	−0.019	0.217
Sex (men vs. women)	−0.056	0.068	−0.026	0.093
Diabetes type (type 2 vs. type 1)	0.042	0.184	0.023	0.146
Etiology of DFUs (neuroischemic vs. ischemic vs. neuropathic)	0.047	0.125	0.032	0.034
Length of hospital stay, days	-	-	0.860	<0.001

DFUs, diabetic foot ulcers.

**Table 4:** Median cost per episode of hospitalization (EUR) by etiology of foot ulcers.

	All Sample N = 1094	2015 N = 280	2016 N = 273	2017 N = 278	2018 N = 263	*p*-Value for Trend
Overall cases with foot ulcers	810.8 (587.6; 1320.9)	815.4 (590.9; 1462.4)	814.6 (603.9; 1286.0)	793.5 (559.8; 1258.2)	816.2 (574.3; 1287.8)	0.332
Neuropathic DFUs						
Type of diabetes						
Type 1	621.5	674.6	627.4	621.3	568.4	0.900
	(531.8; 770.8)	(586.5; 772.6)	(519.9; 780.0)	(512.6; 704.4)	(503.9; 748.5)	
Type 2	635.2	672.4	651.0	593.3	671.1	0.157
	(513.5; 829.9)	(529.9; 858.3)	(503.6; 838.0)	(507.3; 711.8)	(548.0; 848.2)	
*p*-value	0.887	0.990	0.853	0.556	0.488	
By sex						
Women	782.3	840.5	796.2	609.1	625.2	0.271
	(557.5; 960.0)	(579.9; 1023.9)	(554.7; 1163.2)	(560.7; 826.5)	(477.9; 932.0)	
Men	607.5	618.5	596.4	593.5	652.2	0.358
	(509.0; 782.7)	(525.9; 762.2)	(491.8; 789.2)	(504.9; 704.5)	(539.8; 841.7)	
*p*-value	0.001	0.004	0.026	0.360	0.745	
By age groups						
18–40 years	574.0	574.0	4376.5	1627.2	-	0.344
	(498.7; 974.5)	(536.3; 876.8)				
40–65 years	651.0	672.5	691.0	603.8	698.2	0.194
	(527.4; 824.9)	(553.6; 838.7)	(531.3; 823.3)	(506.1; 736.6)	(568.4; 854.6)	
≥65 years	596.4	674.8	554.7	582.7	632.4	0.603
	(497.8; 832.0)	(529.2; 885.8)	(453.7; 806.0)	(511.5; 698.1)	(477.9; 841.7)	
*p*-value	0.603	0.969	0.208	0.396	0.285	
Ischemic DFUs						
Type of diabetes						
Type 1	825.3	1550.9	542.2	881.5	1404.7	0.118
	(530.1; 1216.9)	(1095.2; 1788.1)	(357.7; 829.8)		(1127.5; 1682.0)	
Type 2	1178.4	1464.0	1149.4	1186.8	902.3	0.001
	(708.1; 1740.8)	(932.7; 2220.8)	(768.9; 1671.0)	(669.0; 1839.1)	(607.0; 1665.6)	
*p*-value	0.047	0.819	0.002	0.683	0.466	
By sex						
Women	1158.8	1599.7	1220.2	1084.9	929.8	0.021
	(717.3; 1807.0)	(1031.7; 2033.0)	(779.8; 1839.1)	(624.0; 1933.1)	(622.5; 1400.2)	
Men	1173.7	1373.0	992.9	1199.9	902.3	0.023
	(691.2; 1708.1)	(801.2; 2533.6)	(730.3; 1556.2)	(751.5; 1816.2)	(607.0; 1711.7)	
*p*-value	0.569	0.519	0.203	0.678	0.802	
By age groups						
18–40 years	1237.1	-	-	1088.5	1385.6	1.000
	(1088.5; 1385.6)					
40–65 years	1135.6	1611.3	890.5	1032.9	899.8	0.103
	(664.1; 1927.7)	(857.9; 2658.6)	(630.8; 1676.0)	(667.7; 1721.3)	(653.9; 1715.7)	
≥65 years	1181.8	1409.8	1117.3	1216.9	905.3	0.007
	(713.1; 1693.3)	(939.3; 2025.2)	(788.1; 1584.9)	(713.1; 1857.4)	(602.8; 1602.6)	
*p*-value	0.982	0.527	0.526	0.705	0.723	
Neuroischemic DFUs						
Type of diabetes						
Type 1	729.8	588.2	727.5	1107.8	1136.6	0.143
	(588.2; 1136.6)	(529.0; 692.1)	(569.4; 728.7)	(800.5; 1481.1)	(885.2; 1149.7)	
Type 2	733.1	697.8	708.5	807.4	895.1	0.627
	(596.4; 1232.0)	(582.0; 1085.4)	(598.3; 1109.7)	(609.1; 1434.8)	(579.8; 1418.3)	
p-value	0.708	0.203	0.442	0.548	0.944	
By sex						
Women	964.2	588.2	1161.0	895.5	1382.2	0.425
	(584.0; 1607.1)	(535.4; 1032.9)	(677.1; 2236.4)	(843.9; 1095.0)	(579.8; 1767.1)	
Men	727.2	697.8	708.5	770.9	840.8	0.634
	(596.4; 1136.6)	(589.8; 1036.6)	(590.5; 944.5)	(603.5; 1442.1)	(630.3; 1149.7)	
*p*-value	0.225	0.354	0.123	0.448	0.237	
By age groups						
18–40 years	1200.7	1200.7	-	-	-	-
40–65 years	727.6	697.8	729.1	673.3	1025.9	0.516
	(600.4; 1095.0)	(589.0; 942.6)	(600.2; 884.9)	(609.1; 1274.3)	(705.5; 1149.7)	
≥65 years	798.2	657.6	677.1	843.9	895.1	0.530
	(580.9; 1396.5)	(541.8; 1032.9)	(590.5; 1377.1)	(682.6; 1569.9)	(562.8; 1418.3)	
*p*-value	0.548	0.569	0.783	0.433	0.828	
No specified etiology of the DFUs						
Type of diabetes						
Type 1	1107.5	-	-	1107.5	2722.0	1.000
	(806.5; 3023.1)				(505.4; 4938.6)	
Type 2	646.1	590.2	840.8	607.4	624.5	0.043
	(554.4; 919.8)	(487.5; 739.0)	(608.2; 1120.6)	(507.7; 893.0)	(524.0; 902.0)	
*p*-value	0.312	-	-	0.303	0.650	
By sex						
Women	771.2	525.4	936.0	631.7	774.6	0.006
	(590.8; 936.0)	(493.8; 596.7)	(856.5; 1210.8)	(597.5; 1001.4)	(534.1; 890.2)	
Men	624.5	687.2	660.5	585.5	603.0	0.564
	(511.5; 917.4)	(519.6; 788.3)	(599.4; 1005.2)	(471.5; 890.7)	(517.0; 977.3)	
*p*-value	0.157	0.318	0.035	0.117	0.948	
By age groups						
18–40 years	2938.4	-	-	938.2	4938.7	1.000
	(938.2; 4938.6)					
40–65 years	718.3	652.0	865.1	643.0	771.2	0.471
	(565.4; 955.4)	(535.4; 739.0)	(602.3; 1084.4)	(574.5; 904.1)	(531.0; 922.1)	
≥65 years	632.1	531.8	840.8	602.3	603.0	0.065
	(507.7; 884.1)	(455.0; 733.7)	(637.2; 1143.6)	(504.0; 844.6)	(476.3; 762.7)	
*p*-value	0.122	0.755	0.897	0.420	0.146	

N, number of episodes of hospitalizations; DFUs, diabetic foot ulcers. The Kruskal–Wallis and Mann–Whitney tests were used for the calculation of the *p*-values.

## Data Availability

The data presented in this study are available on request from the corresponding author.

## Supplementary Materials

The following supporting information can be downloaded at: https://www.mdpi.com/article/10.3390/jcm14041248/s1, Table S1: Median length of stay per episode of hospitalization in cases with diabetic foot ulcers; Table S2: Median cost per episode of hospitalization in cases with diabetic foot ulcers.
