# Supplementary material for: The Burden of Diabetic Foot Ulcers on Hospital Admissions and Costs in Romania
Source: J Clin Med. 2025 Feb 13;14(4):1248. doi: 10.3390/jcm14041248 (PMC11855980; doi:10.3390/jcm14041248)
Supplement: Supplementary file 1 [file jcm-14-01248-s001.zip › jcm-3425172-supplementary.pdf]

Supplementary material

Table S1. Median length of stay per episode of hospitalization in cases with diabetic foot ulcers.

	**All samples N=1094**	**2015 N=280**	**2016 N=273**	**2017 N=278**	**2018 N=263**	***p-value* for trend**
**Overall cases with ulceration**	8.0 (6.0; 12.0)	9.0 (7.0; 13.0)	8.0 (6.0; 12.0)	8.0 (6.0; 11.0)	8.0 (6.0; 12.0)	0.075
**Type of diabetes**						
**Type 1**	7.0 (6.0; 11.0)	8.0 (7.0; 10.0)	7.0 (5.5; 8.5)	8.0 (7.0; 11.0)	9.5 (7.0; 12.0)	0.120
**Type 2**	8.0 (6.0; 12.0)	9.0 (7.0; 13.0)	8.0 (7.0; 12.0)	8.0 (6.0; 11.0)	8.0 (6.0; 12.0)	0.057
p-value	0.126	0.276	0.007	0.888	0.549	
**By sex**						
**Women**	9.0 (7.0; 14.0)	10.0 (7.0; 14.0)	10.0 (7.0; 14.0)	8.0 (6.5; 11.5)	8.0 (6.0; 11.0)	0.163
**Men**	8.0 (6.0; 11.0)	8.0 (7.0; 13.0)	8.0 (6.0; 11.0)	8.0 (6.0; 11.0)	8.0 (6.0; 12.0)	0.168
p-value	0.017	0.163	0.004	0.559	0.850	
**By age groups**						
**18–40 years**	9.5 (7.0; 14.0)	7.0 (6.5; 10.5)	11.0	8.0 (6.0; 9.5)	22.5 (18.0; 27.0)	0.177
**40–65 years**	8.0 (6.0; 11.0)	8.0 (7.0; 12.0)	8.0 (6.0; 11.5)	7.0 (6.0; 10.0)	8.0 (6.0; 12.0)	0.142
**≥65 years**	8.0 (6.0; 13.0)	9.0 (7.0; 14.0)	8.0 (6.0; 12.0)	8.0 (6.0; 12.0)	8.0 (6.0; 11.0)	0.228
p-value	0.379	0.428	0.674	0.303	0.122	

N, number of episodes of hospitalizations.

The Kruskal–Wallis and Mann–Whitney tests were used for the calculation of the p-values.

Table S2. Median cost per episode of hospitalization in cases with diabetic foot ulcers.

	**All samples N=1102**	**2015 N=282**	**2016 N=275**	**2017 N=281**	**2018 N=264**	***p-value* for trend**
**Overall cases with foot ulcers**	810.8 (587.6; 1320.9)	815.4 (590.9; 1462.4)	814.6 (603.9; 1286.0)	793.5 (559.8; 1258.2)	816.2 (574.3; 1287.8)	0.332
**Type of diabetes**						
**Type 1**	672.2 (530.9; 886.0)	674.6 (581.1; 863.0)	621.5 (484.2; 770.0)	736.6 (605.2; 994.5)	748.5 (532.1; 1162.8)	0.352
**Type 2**	823.3 (590.3; 1350.6)	832.8 (593.1; 1496.0)	839.5 (617.9; 1354.1)	797.2 (559.4; 1276.7)	816.2 (579.8; 1291.1)	0.182
p-value	0.002	0.052	0.001	0.659	0.653	
**By sex**						
**Women**	919.4 (609.6; 1512.5)	989.9 (593.1; 1623.6)	1042.3 (709.6; 1511.3)	836.6 (591.0; 1527.5)	878.0 (568.0; 1342.9)	0.175
**Men**	773.2 (582.2; 1271.3)	760.2 (589.8; 1400.8)	768.9 (590.5; 1135.0)	731.5 (525.3; 1241.1)	813.4 (586.4; 1281.6)	0.491
p-value	0.001	0.120	<0.001	0.227	0.873	
**By age groups**						
**18–40 years**	1031.5 (574.0; 1200.7)	876.8 (536.3; 1190.2)	974.6	938.2 (647.2; 1013.3)	3162.1 (1385.6; 4938.6)	0.193
**40–65 years**	747.6 (582.1; 1148.2)	733.6 (589.6; 1319.4)	769.1 (588.7; 1135.8)	663.4 (547.5; 1007.7)	816.2 (590.5; 1149.7)	0.179
**≥65 years**	883.9 (592.2; 1452.9)	914.7 (599.8; 1569.2)	896.7 (637.8; 1377.1)	890.8 (578.0; 1521.9)	807.6 (571.0; 1363.1)	0.454
p-value	0.005	0.201	0.135	0.036	0.170	

N, number of episodes of hospitalizations.

The Kruskal–Wallis and Mann–Whitney tests were used for the calculation of the p-values.
